# Rational domestication of a plant-based recombinant expression system expands its biosynthetic range

**DOI:** 10.1093/jxb/erac273

**Published:** 2022-06-20

**Authors:** Mark A Jackson, Lai Yue Chan, Maxim D Harding, David J Craik, Edward K Gilding

**Affiliations:** Institute for Molecular Bioscience, Australian Research Council Centre of Excellence for Innovations in Peptide and Protein Science, The University of Queensland, Brisbane, Queensland, Australia; Institute for Molecular Bioscience, Australian Research Council Centre of Excellence for Innovations in Peptide and Protein Science, The University of Queensland, Brisbane, Queensland, Australia; Institute for Molecular Bioscience, Australian Research Council Centre of Excellence for Innovations in Peptide and Protein Science, The University of Queensland, Brisbane, Queensland, Australia; Institute for Molecular Bioscience, Australian Research Council Centre of Excellence for Innovations in Peptide and Protein Science, The University of Queensland, Brisbane, Queensland, Australia; Institute for Molecular Bioscience, Australian Research Council Centre of Excellence for Innovations in Peptide and Protein Science, The University of Queensland, Brisbane, Queensland, Australia; University of Birmingham, UK

**Keywords:** Asparaginyl endopeptidase (AEP), CRISPR/Cas9, cyclotide, gene editing, insecticide, peptide, plant molecular farming, protease, recombinant, therapeutic

## Abstract

Plant molecular farming aims to provide a green, flexible, and rapid alternative to conventional recombinant expression systems, capable of producing complex biologics such as enzymes, vaccines, and antibodies. Historically, the recombinant expression of therapeutic peptides in plants has proven difficult, largely due to their small size and instability. However, some plant species harbour the capacity for peptide backbone cyclization, a feature inherent in stable therapeutic peptides. One obstacle to realizing the potential of plant-based therapeutic peptide production is the proteolysis of the precursor before it is matured into its final stabilized form. Here we demonstrate the rational domestication of *Nicotiana benthamiana* within two generations to endow this plant molecular farming host with an expanded repertoire of peptide sequence space. The *in planta* production of molecules including an insecticidal peptide, a prostate cancer therapeutic lead, and an orally active analgesic is demonstrated.

## Teaser

Plant molecular farming employs plants as living bioreactors to produce recombinant products. We demonstrate that rationally chosen genome edits can result in plants capable of accumulating previously recalcitrant recombinant peptides.

## Introduction

Plant-based production of therapeutics offers opportunities for production at scale, with a reduced environmental footprint (Fischer and Buyel, 2020). Although plant-based expression systems are relatively underexplored compared with bacterial or mammalian cell recombinant technologies, they offer capacity for post-translational modification that could enhance or expand the utility of recombinant products. Their green advantage arises from their serum-free production and culture at scale, with only inexpensive inputs required such as fertilizer, water, and light without costly infrastructure investment (Walwyn *et al.*, 2015; Nandi *et al.*, 2016). Furthermore, animal cell-free systems negate the threat of zoonotic contaminants, which have derailed product rollouts in the past (Barone *et al.*, 2020). Unlike mammalian or bacterial cell production lines which have the benefit of 50+ years of strain selection (Wuest *et al.*, 2012), plant molecular farming (PMF) is a frontier technology in pharmaceutical production, ripe for genetic and process improvements.

PMF typically employs transient gene expression, which is both rapid and flexible for the production of therapeutic antibodies, enzymes, and vaccines. Indeed, transient expression in *Nicotiana benthamiana* enabled the rapid scale-up and deployment of the first antibody therapy against Ebola (Olinger *et al.*, 2012; Gomez *et al.*, 2021). Plant-based production of a ­seasonal adjustable quadrivalent influenza vaccine is now in phase III clinical trials (Ward *et al.*, 2021b), and the development of SARS-CoV-2 vaccines has matured to the point of being approved by national health authorities (https://covid-vaccine.canada.ca/covifenz/product-details) (Ward *et al.*, 2021a). Although some vaccines, enzymes, and antibodies produced in plant-based systems are on the market or in late-stage trials, there remains a gap in terms of peptide production. There is an opportunity to develop plant systems for producing stabilized therapeutic peptides, by capitalizing on recent advances in the application of plant peptide ligation machinery (Jackson *et al.*, 2020; Rehm *et al.*, 2021).

Some plants natively produce stable head-to-tail cyclic disulfide-rich peptides (cycDRPs), with the best characterized being the three-disulfide-containing cyclotide peptide family (Craik *et al.*, 1999) and the single-disulfide-containing sunflower trypsin inhibitor (SFTI) peptide (Luckett *et al.*, 1999). These peptides are gene encoded and processed from larger precursor proteins as they transit through the plant endomembrane system. The final maturation step is predicted to occur in the vacuole where backbone cyclization is performed by a class of ligase-competent asparaginyl endopeptidases (AEPs), found only in five angiosperm families (Jackson *et al*., 2018, 2020). Once extracted, cyclotides are highly stable, and tolerant of a range of thermal, proteolytic, or chemical insults (Colgrave and Craik, 2004), making them valuable scaffolds for peptide engineering applications (Wang and Craik, 2018). One proven application is for bioactive epitope grafting, which helps stabilize and configure an epitope for improved efficacy and therapeutic half-life (Chan *et al*., 2011, 2016). Thus, cycDRPs are envisaged as customizable vehicles to carry therapeutic sequences.

Although grafted cycDRPs can be produced synthetically at laboratory scale, their production at commercial yields is highly suited to a plant-based system, where both precursor and ligase-capable AEP can be cooperatively stacked. However, in plants generally most suited as biofactory hosts, such as *N. benthamiana*, the endogenous AEPs have not evolved for peptide ligation, but rather retain their ancestral function as hydrolases. Thus, these endogenous AEPs may have a negative influence on the resulting cyclic peptide yield, either from outcompeting and hydrolysing the precursor, or by linearizing any cyclic peptide product formed. Once misprocessed, peptides lack the bond energy required for transpeptidation, thus endogenous AEP activity can have a significant negative impact on the sequence space available for PMF of cyclic peptides.

The development of gene editing tools such as CRISPR (clustered regularly interspaced short palindromic repeats) has enabled targeted genetic improvements of crop species, previously thought impossible (Doudna and Charpentier, 2014). Here we demonstrate the genetic customization of the industrialized PMF crop *N. benthamiana* through rapid and rational ‘domestication’, enabling the accumulation of heretofore unattainable peptides with therapeutic potential in a plant-based system. We describe our simple and effective genomic edits that enable the production of therapeutics to treat prostate cancer, Netherton syndrome, and neuropathic pain. We further show the production of a potent insecticidal peptide naturally produced in garden pea.

## Materials and methods

### Generation of ΔAEP *N. benthamiana* genotype

An AEP knockout line was produced by introducing an array of four gRNA–tRNA repeats, each targeting one of the selected AEPs, into pKIR1.1 which is a CRISPR/Cas9 (CRISPR-associated peptide 9) expression vector carrying the pFAST seed selection system conferring expression of monomeric red fluorescent protein (mRFP) in seeds (Tsutsui and Higashiyama, 2016). Selection of AEP loci to be targeted by CRISPR/Cas9 to introduce mutations was based on the top TBLASTN hits in the version 6.1 Benthgenome annotation set (www.benthgenome.qut.edu.au) when Arabidopsis β-VPE (β-vacuolar processing enzyme; AT1G62710.1) was used as a query (Nakasugi *et al.*, 2014). Targets were further restricted to those showing expression in the Benthgenome Atlas, did not share >90% pairwise identity with any other protein hit to maximize top target diversity, and restricted the maximum number of chosen loci to four to limit pleiotropic effects of reduced AEP function. In pKIR1.1, the crRNA is cloned into the relevant site using *Aar*I, a type IIS restriction enzyme that liberates four bases of unique sequence on both sides of the plasmid backbone. Products were amplified using Phusion polymerase (ThermoFisher Scientific) as per the manufacturer’s protocol in a two-step protocol, except the addition of five cycles at the start of the program where annealing was set to 55 °C for 10 s followed by 30 cycles in a two-step cycle. DMSO at a final concentration of 3% was added to the pGEMT-MOD PCR. The template for pGEMT-MOD was linearized pGEM-T Easy (Promega), and the template for all other reactions was the pTG-mid dsDNA (Supplementary Table S1) fragment synthesized by Integrated DNA Technologies. Products were assembled using gel-purified fragments with the NEBuilder HiFi kit (New England Biolabs) into pGEMT-MOD, and subsequently cloned into pKIR1.1 using *Aar*I in conjunction with T4 ligase (New England Biolabs).

To enable transformation of *N. benthamiana*, the pKIR1.1_ΔAEP construct was transferred into *Agrobacterium tumefaciens* (strain LBA4404) by electroporation. Transformation of *N. benthamiana* leaf discs and regeneration of plants was performed as described by Clemente (2006) with 30 mg l^–1^ hygromycin B used for selection (Pavli *et al.*, 2011). Shoots were rooted in rooting medium as described by Clemente (2006) supplemented with 10 mg l^–1^ hygromycin B. Primary transgenics were acclimatized in a controlled-environment room and grown to maturity where progeny seed was scored for segregation of red fluorescence using a Nikon SMZ18 stereo microscope equipped with a mercury lamp and mRFP filter set. Non-fluorescent seed, devoid of Cas9 expression, were picked up with a moistened 27 gauge needle, stored in a 1.5 ml tube, and, when ready for planting, grown to maturity. Progeny were screened for lesions at AEP loci using cleaved amplified polymorphic sequence (CAPS) markers and Sanger sequencing using gDNA purified with the Jena Science Plant DNA kit. CAPS marker analysis was performed using primers (Supplementary Table S2) followed by digestion of PCR products. Restriction enzymes used were: NbAEP1 (*Alw*I), NbAEP2 (*Psp*GI), NbAEP3 (*Pvu*II), and NbAEP4 (*Sty*I). Amplicons hosting polymorphisms remained undigested compared with digested fragments of wild-type alleles. PCR products from CAPS-positive plants were then selected for Sanger sequencing. A single line (termed ΔAEP) was selected for use in all infiltration experiments. See additional genotyping details at the Dryad Digital Repository (https://doi.org/10.5061/dryad.k6djh9w88).

### Plant cultivation and material

*Nicotiana benthamiana* and ΔAEP plants were cultivated using a hydroponic nutrient system in a controlled plant growth facility as part of the Clive and Vera Ramaciotti Facility for Producing Pharmaceuticals in Plants. The temperature in the growth room was set at 28 °C and plants were grown under 170 µmol m^–2^ s^–1^ of LED illumination in 16 h daylight. (AP67 LED spectra, Valoya Oy, Finland).

### RNA-seq analysis

To determine the global gene expression changes between ΔAEP and wild-type plants, two replicates of each plant were vacuum infiltrated with *A. tumefaciens* carrying pEAQ-eGFP. After 4 d, the plant tissue was sampled, ground to powder in liquid nitrogen, and RNA was isolated using TRIzol reagent (ThermoFisher Scientific) following the standard manufacturer’s protocol. Samples were treated with RNase-free DNase (Ambion), quantified by spectrophotometry, visualized on an agarose gel to check integrity, and submitted for Illumina NextSeq 500 RNA-seq for mRNA input (Australian Genome Research Facility, AGRF). Data are available from the National Center for Biotechnological Information Sequence Read Archive in BioProject PRJNA784697. Reads were trimmed and filtered using Trimmomatic (Bolger *et al.*, 2014), mapped to the Sol Genomics *N. benthamiana* transcript set using Bowtie2 (Langmead and Salzberg, 2012), quantified with kallisto (Bray *et al.*, 2016), and differential gene analysis performed by EBSeq (Leng *et al.*, 2013). Significantly up-regulated or down-regulated genes were identified by a cut off *P*-value of 0.05 and differential genes further refined by a ±2-fold difference between the wild type and ΔAEP. See additional RNA-seq expression details at Dryad.

### Transient expression in *Nicotiana benthamiana* leaf

Peptide expression constructs were ordered as gene blocks (Supplementary Fig. S1) (Integrated DNA Technologies, IDT) with codon usage optimized to *N. benthamiana* according to IDT optimization software. Gateway cloning enzymes (BP and LR Clonase II, Invitrogen) were used to clone the precursor coding sequence initially into the intermediate pDS221 vector (Du *et al.*, 2020), followed by the plant expression vector pEAQ-DEST1 (Sainsbury *et al.*, 2009). For AEP ligase expression, pEAQ-OaAEP1b and pEAQ-CtAEP1 were used and have been previously described (Jackson *et al.*, 2018). For the expression of NbAEPs 1–4, coding sequences were amplified from prepared *N. benthamiana* leaf cDNA and subsequently transferred to pDS221 and to the plant expression vector pEAQ-DEST1 (Sainsbury *et al.*, 2009). All vectors were validated by Sanger sequencing before transfer to *A. tumefaciens* strain LBA4404 for leaf infiltration experiments.

To compare relative peptide yields between *N. benthamiana* and ΔAEP, plants of 5–6 weeks of age were vacuum infiltrated with agrobacterium suspensions containing equal amounts of agrobacterium carrying AEP and precursor peptide constructs as estimated by mixing cultures for a final OD_600_ of 0.5 for each component. Before infiltration, cultures were resuspended in infiltration buffer (10 mM MES pH 5.6, 10 mM MgCl_2_, 100 µM acetosyringone) and allowed to rest for up to 2 h to induce virulence. Plants were harvested 5 d post-infiltration and immediately freeze-dried for storage and peptide extraction.

### Peptide analysis and relative quantification

Freeze-dried tissue was ground to a fine powder using a GenoGrinder (SPEX SamplePrep). Peptides were extracted using a 50% (v/v) acetonitrile, 1% (v/v) formic acid solution at a ratio of 20 µl mg^–1^ of tissue DW. Peptides were extracted overnight with gentle mixing before centrifugation to pellet insoluble material. A 10 µl aliquot of the peptide-containing supernatant was then mixed with an equal volume of an unrelated control peptide (GCCSDPRCNYDHPEICGGAAGN) and a further 80 µl of an 80% (v/v) acetonitrile, 1% (v/v) formic acid solution. For matrix-assisted laser deionization-time of flight (MALDI-TOF-MS), this diluted and spiked peptide mix was mixed 1:1 with a α-cyano-4-hydroxycinnamic acid [5 mg ml^–1^ in 50% acetonitrile/0.1% trifluoroacetic acid (TFA)/5 mM (NH_4_)H_2_PO_4_] solution before being spotted and dried on a MALDI plate. MALDI-TOF-MS spectra data was acquired using a 5800 MALDI-TOF MS (AB SCIEX, Canada) operated in reflector positive ion mode. For relative yield determination, the isotope cluster area corresponding to the peptide of interest was normalized to that obtained for the internally spiked peptide control.

### Scale-up production of Pa1b and purification

To produce and purify *N. benthamiana* derived Pa1b peptide for cytotoxicity assessment, 10 ΔAEP plants were infiltrated by submerging whole plants in an agrobacterium suspension and applying and releasing a vacuum. At 5 d post-infiltration, plant leaves were sampled, freeze-dried, and ground to fine powder using a GenoGrinder. Peptides were extracted overnight using 50% (v/v) acetonitrile, 1% (v/v) formic acid at a ratio of 50 µl mg^–1^ of tissue DW. After centrifugation to pellet insoluble material, the peptide-containing supernatant was again freeze-dried and resuspended in 10% (v/v) acetonitrile, 1% (v/v) formic acid before solid-phase extraction (SPE) using a Phenomenex Strata C18-E SPE cartridge with 10 g resin capacity. The eluted 10–50% (v/v) acetonitrile, 1% (v/v) formic acid fraction was then pooled, lyophilized, and reconstituted in 10% (v/v) acetonitrile, 0.1% (v/v) TFA in preparation for HPLC on a semi-preparative Phenomenex Jupiter C18 RP-HPLC column (250 m×10 mm, 5 µm particle size) connected to a Shimadzu LC-20AT pump system (Shimadzu Prominence). The purity of peptides was checked by MALDI-TOF-MS and analytic HPLC using a C18 column (Phenomenex, Jupiter® 5 µm, 300 Å, 150 × 2.0 mm).

### Cytotoxicity assessment of recombinantly produced Pa1b

*Spodoptera frugiperda* (Sf9) insect cells were cultured in ESF 921 medium at 27 °C, 5% CO_2_ in 75 cm^2^ flasks until 80% confluent. Cells were plated into 96-well tissue culture plates (100 μL, 10 000 cells per well) and grown for 24 h prior to treatment. Peptides were added in triplicate with final concentrations ranging from 0.002 μM to 50 μM, while control wells were incubated with 1% Triton X-100 and medium only. The cells were further incubated for 24 h at 27 °C, 5% CO_2_. A 10 μL aliquot of MTT [3-(4,5-dimethylthiazol-2-yl)-2,5-diphenyltetrazolium bromide, 5 mg ml^–1^ in phosphate-buffered saline (PBS)] was added to each well to a final concentration of 0.5 mg ml^–1^ and were incubated for 3 h at 27 °C, 5% CO_2_. Supernatants were removed, and the insoluble formazan crystals were resuspended in 100 μL of DMSO. The plate was shaken at room temperature for 10 min to fully dissolve the formazan crystals, and the absorbance of the solutions was measured at 600 nm on a Tecan Infinite M1000Pro plate reader.

### Statistical analysis

One-way ANOVA was performed using GraphPad Prism version 9.00 for Mac OS (GraphPad Software, La Jolla, CA, USA).

## Results

### Cyclic SFTI-1 therapeutic peptide candidates harbouring Asn residues are inefficiently produced in *N. benthamiana*

SFTI-1 is a 14 amino acid cyclic peptide naturally produced in sunflower seed, and is a favoured scaffold for peptide ­engineering applications (de Veer *et al.*, 2021). Previously, using *N. benthamiana* as a host for transient gene expression, we demonstrated the successful *in planta* production of SFTI-1 as well as a variant displaying low picomolar inhibition of the human serine protease plasmin (Jackson *et al.*, 2019; Swedberg *et al.*, 2019). In the same study, we attempted to improve the cyclization yield by substituting the Asp residue of SFTI-1 with an Asn, which we predicted, for some AEPs, could improve the cyclization efficiency. However, unlike the native SFTI-1, SFTI-1_N could not be produced *in planta*, which led to the hypothesis that the Asp–Asn residue exchange is detrimental to the stability of the peptide *in planta*.

We first aimed to determine if this problem of instability is more broadly applicable to other SFTI-1 therapeutic candidates, including examples proposed as potential leads for the treatment of prostate cancer. To approach this question, we assembled three additional expression constructs, two encoding SFTI-1 variants designed to inhibit human kallikrein 4 (SFTI-1_KLK4_D and SFTI-1_KLK4_N) (Swedberg *et al.*, 2011; Riley *et al.*, 2019) and one encoding a kallikrein 5 inhibitor (SFTI-1_KLK5_N) (de Veer *et al.*, 2016). The two kallikrein 4 inhibitors were identical, apart from an Asp–Asn exchange at the cyclization residue. This single residue change (Asp–Asn) was previously demonstrated to confer a 125-fold increase in potency (*K*_i_=0.04 nM) and enhanced selectivity over off-target serine proteases (Swedberg *et al.*, 2011). Thus, of the two peptides, SFTI-1_KLK4_N represents the preferred candidate for plant-based production. The kallikrein 5 inhibitor was likewise chosen as a good test peptide, where the best performing peptide harboured an Asn residue at the AEP processing site and was most potent (*K*_i_=4.2 nM) (de Veer *et al.*, 2016).

**Fig. 1.**
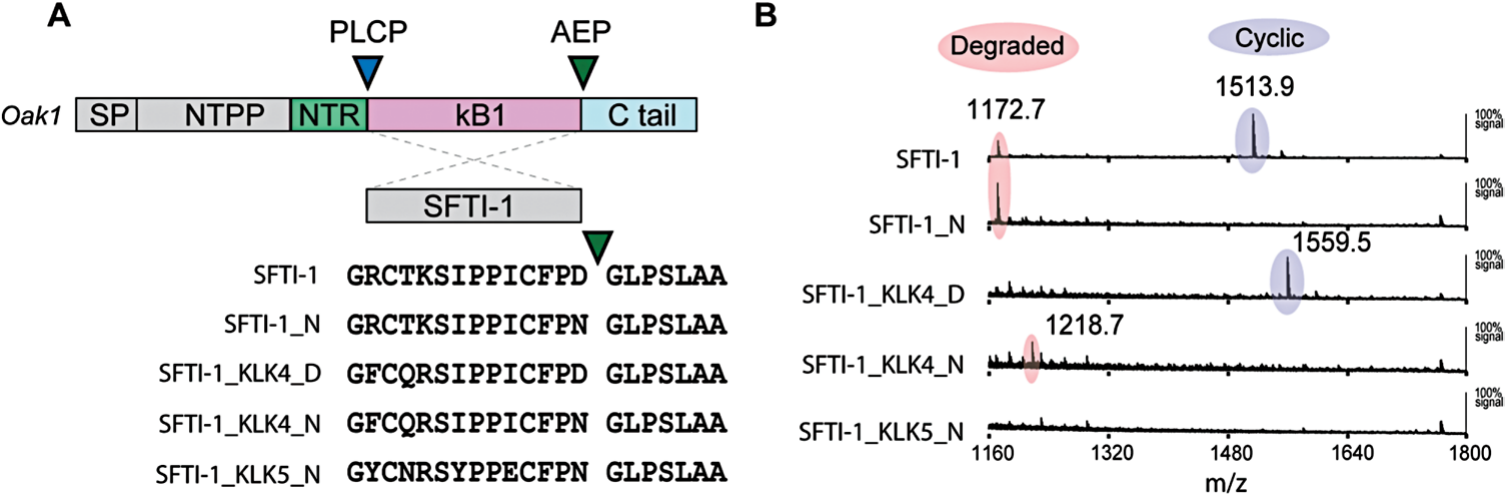
Therapeutic peptide expression in *N. benthamiana*. (A) SFTI-1, SFTI-1_N, and therapeutic peptide candidates were prepared for plant-based expression by insertion of the peptide-coding sequence into the *Oak1* gene, replacing the sequence domain for the cyclotide kB1. The processing of the engineered SFTI-1 precursors is predicted to be controlled by a papain-like cysteine protease (PLCP) and an asparaginyl endopeptidase (AEP) at the N- and C-terminus, respectively. The Oak1 signal peptide (SP), N-terminal propeptide (NTPP), and N-terminal repeat (NTR) sequence ensure that the precursor enters the endomembrane system with targeting towards the vacuole. (B) Co-expression of engineered SFTI-1 precursors with the AEP ligase OaAEP1b in *N. benthamiana* resulted in MALDI-MS detection of either degraded peptides (shaded in peach) or cyclic peptides (shaded in purple).

For expression *in planta*, we assembled SFTI-1 peptide expression constructs by incorporating back-translated SFTI- therapeutic candidates into the Oak1 precursor framework, replacing the sequence encoding the cyclotide kB1, and retaining the papain-like cysteine protease (PLCP) cleavage site (Rehm *et al.*, 2019) (Fig. 1A). To ensure high-level transient expression in *N. benthamiana*, we assembled each designer precursor peptide gene and AEP ligase gene into the pEAQ vector system, which allowed for co-infiltration (Sainsbury *et al.*, 2009). As previously demonstrated for SFTI-1 (Jackson *et al.*, 2019), infiltration of *N. benthamiana* leaf with agrobacterium harbouring precursor gene constructs alone produced no detectable cyclic peptide masses as assessed using MALDI-TOF-MS. However, upon co-infiltration with agrobacterium harbouring the expression vector for ligase-competent AEP (OaAEP1b), backbone cyclic masses were observed for native SFTI-1 and SFTI-1_KLK4_D (Fig. 1B). For the remaining three peptides, each containing an Asn at the cyclization residue, no cyclic masses were evident, suggesting that these peptides are highly unstable in *N. benthamiana* leaf cells.

### A quadruple AEP knockout depletes competing AEP activity with minimal deleterious phenotypic effects

As the minimal change required to induce *in planta* instability of SFTI-1 and the tested variants was a single Asp–Asn substitution, we hypothesized that endogenous AEP activity at Asn residues is problematic. To test this hypothesis, we simultaneously produced lesions in four *N. benthamiana* AEP sequences using CRISPR/Cas9. The chosen loci Niben101Scf04675g08014.1, Niben101Scf04539g04014.1, Niben101Scf18356g00003.1, and Niben101Scf04779g01004.1 were given the names NbAEP1, NbAEP2, NbAEP3, and NbAEP4, respectively.

AEP knockout lines were produced by introducing an array of four gRNA–tRNA repeats, each targeting one of the selected AEPs, into pKIR1.1, which is a CRISPR/Cas9 expression vector carrying the pFAST seed selection system conferring expression of mRFP in seeds (Tsutsui and Higashiyama, 2016). A hemizygous primary transformant was allowed to self, with the resulting seed negatively selected for mRFP expression, thus giving rise to a series (*n*=7) of genome-edited genotypes in the T_1_ generation that were no longer carrying the pKIR1.1 CRISPR/Cas9 cassette. Sites targeted by the crRNA array have restriction enzyme sites present near the protospacer adjacent motif (PAM) end of the crRNA to enable the use of the site as a CAPS marker (Supplementary Fig. S2). In this way, transgene-free T_2_ genotypes carrying some form of mutation at the target loci were identified.

Seven plant lines were selected from the T_2_ population for further analysis. Amplicons from gDNA containing target sites were examined by Sanger sequencing to categorize mutations present. The lesions identified in the T_2_ led to non-silent point mutations, frameshifts, and premature stop codons at the loci targeted. However, some combination of bi-allelic states were observed for all NbAEPs in the population of selected plants, highlighting the need to select for a quadruple homozygous individual. Plants were allowed to self, and quadruple mutants homozygous at the chosen loci were identified in the T_4_ generation by CAPS screening and again by validating allelic states with Sanger sequencing of amplicons (Supplementary Fig. S2). In this way, a single line harbouring mutations in all four targeted AEPs was selected and named ΔAEP. The ΔAEP genotype is homozygous for an 18 bp deletion encompassing the splice acceptor site of exon 4 in NbAEP1, insertion of a single nucleotide at position 388 of the NbAEP2 coding sequence, a single nucleotide insertion at position 544 of the NbAEP3 coding sequence, and a missense mutation at position 538 of the NbAEP4 coding sequence.

RNA-seq analysis was performed on T_5_ ΔAEP and wild-type plants to further validate the structure of the resulting NbAEP transcripts and observe global gene expression changes between genotypes in a transient expression experiment. Both ΔAEP and wild-type plants were infiltrated with pEAQ-eGFP and samples of infiltrated tissue taken for RNA-seq (*n*=2 per genotype). Reads of ΔAEP were mapped against the Sol Genomics *N. benthamiana* Niben101 transcript set and visualized to validate changes in NbAEP mRNA. The ΔAEP NbAEP1 allele produced transcripts with disrupted exon splicing at exon 4 as seen by a lack of read coverage at the crRNA target site (Supplementary Fig. S2), thus confirming the NbAEP1 allele present in ΔAEP as null. Expression levels were estimated, and this revealed that NbAEP1, NbAEP2, and NbAEP3 were significantly down-regulated in ΔAEP (>2.9-fold down-regulated, *P*-values <0.007) (SupplementaryTable S3). NbAEP4 exhibited very low expression in both genotypes such that it did not result in mapped reads above the threshold for abundance estimation. In addition to the down-regulated AEP transcripts in ΔAEP, we observed one other transcript that was significantly down-regulated <2-fold, and 13 transcripts significantly up-regulated >2-fold (Supplementary Table S3). In summary, our ΔAEP genotype resulted in minimally significant perturbations to the agrobacterium-infiltrated wild-type *N. benthamiana* transcriptome. Furthermore, ΔAEP plants were morphologically indistinguishable from the wild type. A simple biomass experiment using a randomized plot design under artificial light and growth conditions failed to reveal significant differences in biomass between genotypes [mean DW per 4-week-old plant (*n*=10): wild type 4.256 ± 0.371 g, ΔAEP 4.281 ± 0.665 g].

**Fig. 2.**
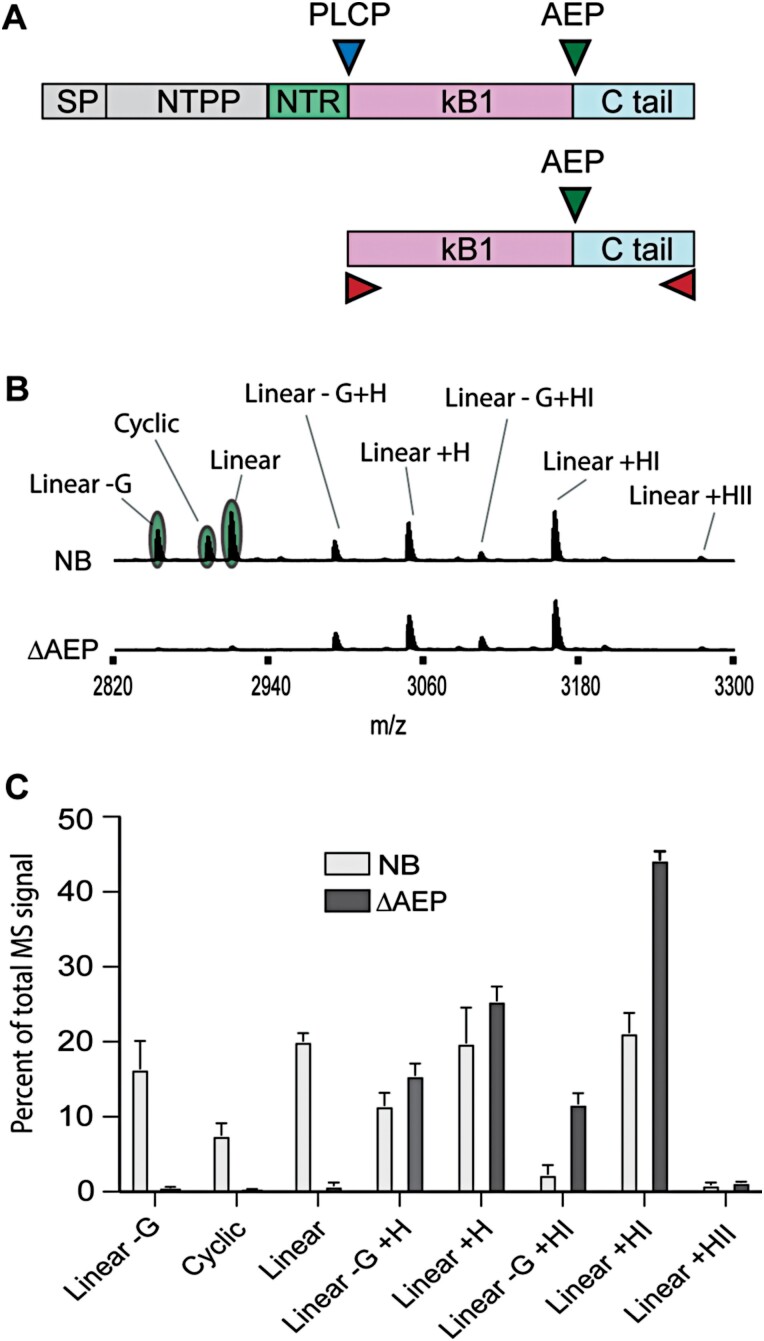
Comparison of recombinant peptide endogenous processing in wild-type versus ΔAEP *N. benthamiana*. (A) The Oak1 precursor is predicted to be processed initially at the N-terminus by a papain-like cysteine protease (PLCP) (blue triangle), followed by asparaginyl endopeptidase (AEP) processing (green triangle) at the C-terminus. Without the co-expression of an AEP ligase, endogenous AEPs that prefer hydrolysis over ligation will compete for the expressed substrate with amino- and carboxypeptidases (red triangles). (B) MALDI-TOF-MS analysis (representative) of *Oak1_HIIAA* expression in wild-type *N. benthamiana* (NB) alongside the ΔAEP gene-edited accession. Only the signals highlighted in green can be attributed to endogenous AEP processing, with the remainder representing carboxypeptidase C-terminal processing events. (C) Mean and SD (*n*=3) of the individual kB1 identified masses as a percentage of the total kB1 peptide MS signal detected by MALDI-TOF-MS.

To determine the effectiveness of our AEP knockout strategy in transient expression of peptides, we first assessed and compared processing of a modified cyclotide precursor gene *Oak1_HIIAA* in wild-type and ΔAEP plants (Fig. 2). It was demonstrated previously that *Oak1* expression in *N. benthamiana* without co-expression of a helper AEP ligase results in accumulation of linear, linear extended, linear truncated, and a small MS signal representing cyclic kB1 (Saska *et al.*, 2007; Gillon *et al.*, 2008). Of these, only the cyclic, full-length linear, and linear minus a Gly at the N-terminus can be attributed to AEP processing, with the remainder representing processing by carboxypeptidases that compete for the substrate (Fig. 2A). For our analysis, to ensure that MALDI-TOF-MS could be used to differentiate all predicted processed products, we modified the C-terminal propeptide (CTPP) residues of Oak1 to HIIAA from the natural GLPSLAA, as with the latter construct it is impossible to differentiate the linear kB1 mass from a linear form harbouring a truncated N-terminal Gly and a C-terminal extended Gly. Expression of *Oak1_HIIAA* in wild-type *N. benthamiana* resulted in MS signals (as a percentage of total kB1-related signals) of 7.6 ± 2.7% for cyclic, 20.1 ± 1.8% for linear, and 16.4 ± 6.4% for linear Gly, with the remaining ~58% of signal being for peptides carrying C-terminal extensions with or without the N-terminal truncation event. In contrast, expression within the ΔAEP line produced almost no detectable signal for cyclic, linear, or linear Gly peptide, with ~100% of the signal representing non-AEP processed forms (Fig. 2B, C). These results clearly demonstrate the effectiveness of our AEP knockout strategy to remove interfering endogenous AEP activity.

### AEP depletion expands accessible peptide sequence space enabling the expression of cyclic therapeutic peptide leads

**Fig. 3.**
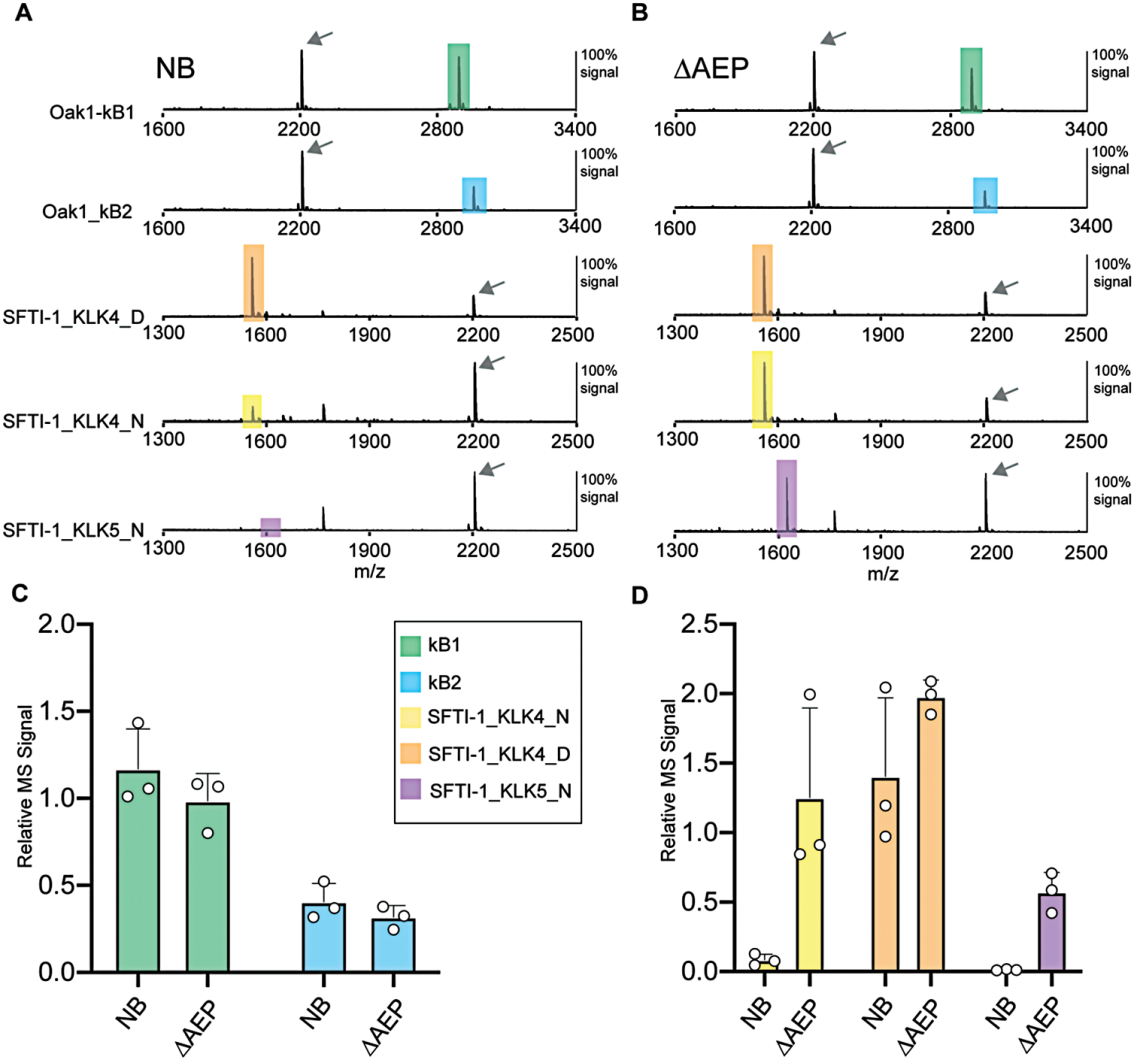
Comparative expression of cyclic peptides in wild-type versus ΔAEP *N. benthamiana*. Representative MALDI-TOF-MS of cyclic peptides accumulated in (A) wild-type *N. benthamiana* (NB) and (B) the ΔAEP accession in transient co-expression with OaAEP1b. Test cyclotides are encoded in the Oak1 precursor that natively contains kB1, or with kB1 swapped for kB2, a cyclotide that ends with an Asp residue. MS signals for cyclic peptides are highlighted to match the colours in (C) and (D). An arrow indicates the MS signal for the internally spiked peptide control that served to normalize MS signals for relative quantification. (C) Mean and SD (*n*=3) of relative kB1 and kB2 MS signals detected in crude peptide extracts of infiltrated *N. benthamiana* (NB) and the ΔAEP accession. (D) Mean and SD (*n*=3) of relative SFTI-1_KLK4_N, SFTI-1_KLK4_D, and SFTI-1_KLK5_N MS signals detected in crude peptide extracts of infiltrated *N. benthamiana* (NB) and the ΔAEP accession.

Having established an *N. benthamiana* plant line with reduced endogenous AEP activity, we next tested our hypothesis that reduced endogenous AEP activity would have a positive influence on the yield of Asn-containing SFTI-1_KLK4_N and SFTI-1_KLK5_N. We repeated infiltration experiments and compared relative yields obtained from wild-type and ΔAEP plants. As previously demonstrated, close to no MS signal for cyclic SFTI-1_KLK4_N and SFTI-1_KLK5_N could be detected in infiltrated wild-type plants. In contrast, MS signals for cyclic SFTI-1_KLK4_N and SFTI-1_KLK5_N were readily detectable when constructs were infiltrated into the ΔAEP genotype (Fig. 3A, B), representing a considerable breakthrough. For the expression of cyclotides kB1 and kB2, relative yields were slightly reduced in ΔAEP plants. A ­subsequent test of another AEP ligase CtAEP1 in ΔAEP plants gave a similar result (Supplementary Fig. S3). Further clarification of the negative effect in the ΔAEP genotype for kB1 and kB2 substrates is planned, with a reduced transactivation of AEP ligase probably playing a role.

### Cyclotides are more resistant than SFTI-1 molecules to endogenous AEP activity

**Fig. 4.**
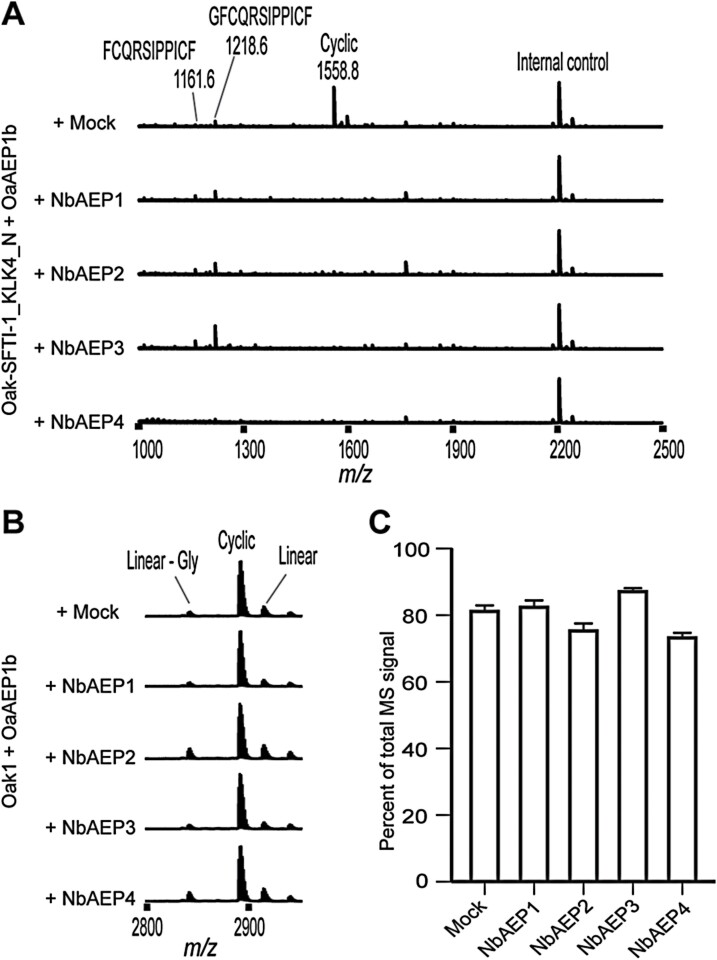
Rescue of AEP function in ΔAEP *N. benthamiana.* Representative MALDI-TOF-MS of (A) cyclic SFTI-1_KLK4_N and (B) cyclic kB1 accumulation in the ΔAEP accession upon co-expression of peptide precursor, AEP ligase, and NbAEP genes. (C) Mean and SD (*n*=3) of the percentage of MS signal representing cyclic kB1 upon co-expression.

In common with all plant species, cyclotide producers encode AEPs as a multigene family with isoforms differing in their propensity for peptide ligation (Serra *et al.*, 2016; Harris *et al.*, 2019). Thus cyclotides, which naturally co-locate with AEPs in vegetative cell vacuoles (Conlan *et al.*, 2011; Slazak *et al.*, 2016), may have evolved structures that are more resistant to hydrolytic AEPs than for the Asn-containing SFTI-1 peptides tested in this study. To gain further insight into this, we set up an infiltration experiment in our ΔAEP plant where we co-expressed AEP ligase (OaAEP1b), individual NbAEPs, and the precursor genes of Oak1 or SFTI-1_KLK4_N (Fig. 4). For SFTI-1_KLK4_N, co-expression of any of the four tested *N. benthamiana* AEPs resulted in a complete elimination of cyclic product formation, as is observed in wild-type plants (Fig. 4A). In contrast, Oak1 processing appears to be only moderately affected, with cyclic kB1 predominating in the MS profile, irrespective of NbAEP overexpression (Fig. 4B, C).

### Bioactive linear peptide accumulation

**Fig. 5.**
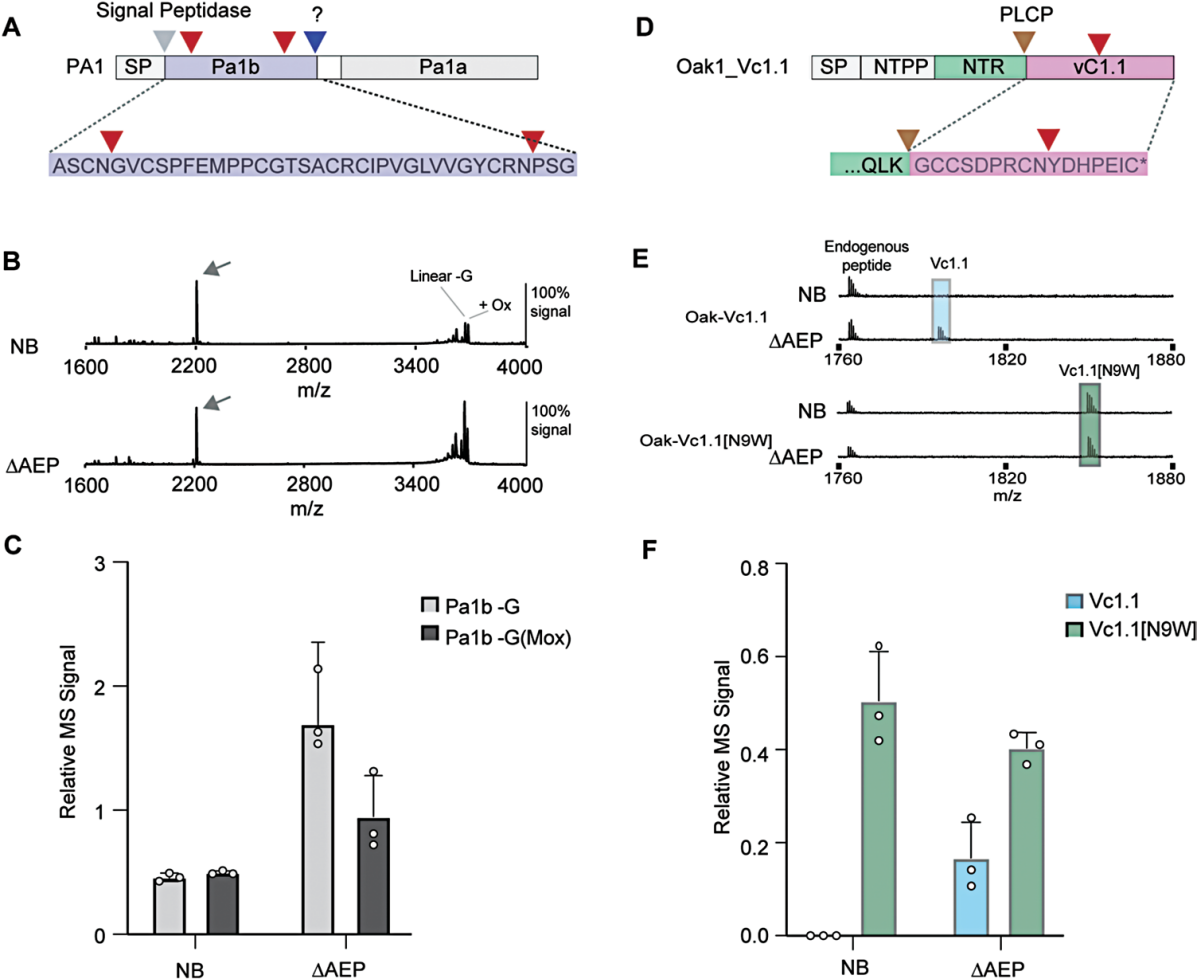
Expression of two recombinant peptides negatively affected by endogenous AEP activity. (A) The insecticidal peptide Pa1b is encoded by the pea albumin 1 gene (*PA1* gene) that additionally codes for the larger PA1a domain of unknown function. The Pa1b N-terminus is predicted to be released via a signal peptide (SP) cleavage event during co-translation into the endoplasmic reticulum. The protease responsible for the release of the Pa1b C-terminus is unknown. Two internal Asn sites are present (red triangles) that may represent putative AEP processing sites. (B) Representative MALDI-TOF-MS of crude peptide extracts prepared from infiltrated leaves of *N. benthamiana* (NB) and ΔAEP. Indicated by an arrow is the MS signal for the internally spiked peptide control that served to normalize MS signals for relative quantification. (C) Mean and SD (*n*=3) of the relative MS signal representing Pa1b-G and Pa1b-G(Mox) peptides. (D) The conotoxin Vc1.1 and its analogue Vc1.1[N9W] were prepared for plant-based expression by insertion of peptide-coding sequence into the *Oak1* gene, replacing both the cyclotide kB1 domain and the C-terminal tail. Processing was predicted to be controlled by a papain-like cysteine protease (PLCP) (brown arrow). One internal Asn site (indicated by a red arrow) is present in Vc1.1, substituted for a Trp in Vc1.1[N9W]. (E) Representative MALDI-TOF-MS of crude peptide extracts prepared from infiltrated leaves of *N. benthamiana* (NB) and ΔAEP. (F) Mean and SD (*n*=3) of the relative MS signal representing Vc1.1 and Vc1.1[N9W].

Having shown that we could improve the *in planta* yield of cyclic SFTI-1 therapeutic leads, we next aimed to determine if the same could be true for bioactive peptides harbouring internal Asn sites. We first chose to test expression of the pea albumin-1 gene (*PA1*) that encodes the 37 amino acid disulfide-rich insecticidal peptide Pa1b (Higgins *et al.*, 1986). This peptide is of high interest for development as it represents the first ever peptide that specifically inhibits insect vacuolar proton pumps (Chouabe *et al.*, 2011). Although produced naturally in many legumes, the development of Pa1b as a commercial insecticide would benefit from an expression platform allowing the rapid testing of variants for improved yield and potency, and with the capacity for scaled up production (Eyraud *et al.*, 2013). Pa1b has two internal Asn sites that represent putative processing sites of endogenous AEPs, thus Pa1b represents a good peptide to test for yield improvements in our ΔAEP *N. benthamiana* line devoid of AEP activity (Fig. 5A). Similar to the *PA1* expression results reported by Eyraud *et al.* (2013), we found that *PA1* expression in *N. benthamiana* results in a number of processed forms, with the predominant signals representing Pa1b with the C-terminal Gly37 removed, with or without oxidation (plus 16 Da) of the Met residue. We found this similar pattern of processing in both wild-type and ΔAEP plants; however, relative yields were calculated to be ~3.7-fold and ~1.9-fold higher in ΔAEP plants for Pa1b-Gly and Pa1b-Gly+Met^ox^, respectively (Fig. 5B, C). By scaling up plant infiltration, Pa1b yield was calculated at ~0.2 mg g^–1^ tissue DW at 95% purity, and the plant-derived peptide was shown to be cytotoxic to Sf9 cells, with a CC_50_ of 13.58 nM (Supplementary Fig. S4).

As an example of a non-plant peptide with therapeutic potential, we prepared constructs (Fig. 5D) for the expression of the 16 amino acid two-disulfide-containing conotoxin Vc1.1, which is a lead compound for the treatment of neuropathic pain (Clark *et al.*, 2010). Native Vc1.1 harbours one internal Asn site, but structure–activity studies have identified that this residue can be substituted with a Trp with minimal changes to activity (Yu *et al.*, 2013). This allowed us to test and compare the expression of the Asn-containing Vc1.1 and Vc1.1[N9W] in wild-type plants and in our ΔAEP line. Of the two variants, only Vc1.1[N9W] could be detected in wild-type plants, indicating that this Asn processing site is liable to processing (Fig. 5E, F). In our ΔAEP line, both Vc1.1 and Vc1.1[N9W] could be detected, suggesting that reducing endogenous AEP levels is beneficial for the accumulation of linear Vc1.1, in a similar fashion to Pa1b.

## Discussion

The uptake of *N. benthamiana* for industrial PMF is gaining momentum, with commercial entities employing *N. benthamiana* as the preferred biofactory host in Africa, North America, and Europe. In particular, the ‘Lab’ strain, derived from a wild *N. benthamiana* accession, which naturally carries a genetic lesion that reduces transgene silencing, has been the dominant accession for transient expression-based production for PMF (Bally *et al.*, 2018). Genetic customization reported to date of the ‘Lab’ accession has been limited to the alteration of the glycosylation pathway, to humanize recombinant glycoproteins (Jansing *et al.*, 2019). Altering *N. benthamiana* to create new genotypes exhibiting expanded expression capability represents great untapped potential. The work presented here demonstrates that potential, made possible by multiplex CRISPR gene editing, whereby knocking out four endogenous AEPs enabled the production of previously non-producible peptide products.

The *N. benthamiana* ΔAEP genotype is similar to the quadruple AEP mutant described from Arabidopsis in that it does not exhibit a deleterious phenotype in controlled growth environment settings (Gruis *et al.*, 2004). In Arabidopsis, the quadruple AEP knockout genotype is however impaired in programmed cell death and in the hypersensitive response when challenged with *Pseudomonas syringae*, indicating a role for AEPs in pathogen response (Rojo *et al.*, 2004). Further roles for AEPs include storage protein processing (Shimada *et al.*, 2003), and separation of the endosperm from the testa during seed development, demonstrated in Arabidopsis (Nakaune *et al.*, 2005). In our *N. benthamiana* ΔAEP genotype, no deleterious phenotypes were observed in controlled growth conditions, and biomass was unaffected. In field conditions or in controlled environments where pathogen infection may occur, the ΔAEP genotype might, however, exhibit greater ­susceptibility, and further exploration of pathogen susceptibility due to reduced AEP function is warranted.

The proteolytic stability of recombinant proteins produced in plants remains a significant challenge. It is clear that there is not a one size fits all approach to improving stability, with larger recombinant proteins possibly degraded by numerous proteases *in planta* or during the extraction phase. Strategies developed to counter proteolytic interference have included the co-expression of protease inhibitors (Grosse-Holz *et al.*, 2018; Ma *et al.*, 2021), the re-engineering of protease-susceptible residues, and in directing subcellular compartmentation to protein-friendly organelles (Streatfield, 2007). In the case of therapeutic peptide production in plants, our approach for enhancing recombinant peptide stability involves co-expression of a ligase-capable AEP with a peptide substrate amenable to AEP-mediated backbone cyclization (Poon *et al.*, 2018). With their lack of termini, backbone cyclic peptides have been demonstrated to have improved stability, both in human serum stability assays (Wong *et al.*, 2012; Ganesan *et al.*, 2021; Muratspahić *et al.*, 2021) and during accumulation in plant cells (Jackson *et al.*, 2019). Ligase-type AEPs, essential for backbone cyclization, however, have only been identified from cyclotide-producing plant species (Jackson *et al.*, 2020), thus they must be exogenously co-expressed in biofactory hosts such as *N. benthamiana*.

Through CRISPR/Cas9 gene editing, we have shown here that the reduction of endogenous AEP activity in *N. benthamiana* positively influences the accumulation of the Asn-containing cyclic SFTI-1 therapeutic peptide candidates [SFTI-1_KLK4_N (Swedberg *et al.*, 2011; Riley *et al.*, 2019) and SFTI-1_KLK5_N (de Veer *et al.*, 2016)] (Fig. 3). This interference by endogenous AEPs appears particularly problematic for Asn-containing SFTI-1 molecules, and not for cyclotides, which have probably evolved to remain resistant to AEP-mediated hydrolysis (Fig. 4). Mechanistically, breakdown of an Asn-containing SFTI-1 molecule may occur through either endogenous AEPs outcompeting the transgene-derived AEP ligase, or by endogenous AEP activity re-cleaving any cyclic peptide generated. We further demonstrate improved production levels upon transient expression in the ΔAEP genotype of the linear peptides Vc1.1 and Pa1b, that each carry internal Asn residues (Fig. 5). Of particular interest was the 1.9 to 3.7-fold increase in the relative yield of Pa1b-Gly+Met^ox^ and Pa1b-Gly, respectively, when compared with expression in wild-type *N. benthamiana*. Interestingly, using MALDI-TOF-MS, no intermediate Asn-specific cleavage products were detectable, suggesting rapid hydrolysis of peptide fragments after the primary AEP cleavage event. Thus, this inefficiency in Pa1b production in wild-type *N. benthamiana* could not be forecast, suggesting that many other small Asn-containing peptides could unknowingly benefit from production in the ΔAEP genotype. Similarly, when the Vc1.1 peptide was expressed in wild-type *N. benthamiana*, no peptide at all could be detected, which could be attributed to either protease-mediated instability or inefficient peptide folding. Only by expressions in the ΔAEP genotype could we identify that AEP hydrolysis was limiting the accumulation of the Vc1.1 peptide. Likewise, other non-accumulating PMF products containing internal protease sites may benefit from select knockout of offending proteases.

Here we have demonstrated genome editing as a valuable strategy to enable the accumulation of cyclic bioactive peptides in the framework of PMF. In reducing endogenous AEP activity of *N. benthamiana*, we have demonstrated an expansion of the repertoire of peptides that this favoured biofactory plant can achieve. The targeting of interfering proteases is an approach that may be applied to other recombinant products that may have failed previously. The work thus potentially has broad implications for the efficient and economical recombinant production of therapeutic and insecticidal peptides.

## Supplementary data

The following supplementary data are available at *JXB* online.

Fig. S1. Gene sequences ordered as dsDNA gene blocks used in this study.

Fig. S2. crRNA target sites for NbAEP1, 2, 3, and 4, CAPS marker sites, and resulting mutations for the ΔAEP genotype.

Fig. S3. CtAEP1 (butelase-1) activity assessment in the ΔAEP accession.

Fig. S4. Purity and cytotoxicity assessment of Pa1b.

Table S1. Primers used in construction of the crRNA array.

Table S2. Primers for genotyping genome-edited plants.

Table S3. Genes significantly up-regulated and down-regulated >2-fold.

erac273_suppl_Supplementary_Material

## Data Availability

The RNA-seq data comparing gene expression between wild-type and ΔAEP *N. benthamiana* are available through the National Center for Biological Information-Sequence Read Archive BioProject PRJNA784697. Data used to create figures and perform analyses are available online in the Dryad repository: https://doi.org/10.5061/dryad.k6djh9w88; Jackson *et al.* (2022).

## References

[CIT0001] Bally J , Jung H, Mortimer C, Naim F, Philips JG, Hellens R, Bombarely A, Goodin MM, Waterhouse PM. 2018. The rise and rise of *Nicotiana benthamiana*: a plant for all reasons. Annual Review of Phytopathology 56, 405–426. 10.1146/annurev-phyto-080417-050141 30149789

[CIT0002] Barone PW , Wiebe ME, Leung JC, et al. 2020. Viral contamination in biologic manufacture and implications for emerging therapies. Nature Biotechnology 38, 563–572. 10.1038/s41587-020-0507-2 32341561

[CIT0003] Bolger AM , Lohse M, Usadel B. 2014. Trimmomatic: a flexible trimmer for Illumina sequence data. Bioinformatics 30, 2114–2120. 24695404 10.1093/bioinformatics/btu170 PMC4103590

[CIT0004] Bray NL , Pimentel H, Melsted P, Pachter L. 2016. Near-optimal probabilistic RNA-seq quantification. Nature Biotechnology 34, 525–527. 10.1038/nbt.3519 27043002

[CIT0005] Chan LY , Craik DJ, Daly NL. 2016. Dual-targeting anti-angiogenic cyclic peptides as potential drug leads for cancer therapy. Scientific Reports 6, 35347. 27734947 10.1038/srep35347 PMC5062114

[CIT0006] Chan LY , Gunasekera S, Henriques ST, Worth NF, Le SJ, Clark RJ, Campbell JH, Craik DJ, Daly NL. 2011. Engineering pro-angiogenic peptides using stable, disulfide-rich cyclic scaffolds. Blood 118, 6709–6717. 22039263 10.1182/blood-2011-06-359141

[CIT0007] Chouabe C , Eyraud V, Da Silva P, Rahioui I, Royer C, Soulage C, Bonvallet R, Huss M, Gressent F. 2011. New mode of action for a knottin protein bioinsecticide pea albumin 1 subunit b (PA1b) is the first peptidic inhibitor of V-ATPase. Journal of Biological Chemistry 286, 36291–36296. 21890633 10.1074/jbc.M111.281055 PMC3196078

[CIT0008] Clark RJ , Jensen J, Nevin ST, Callaghan BP, Adams DJ, Craik DJ. 2010. The engineering of an orally active conotoxin for the treatment of neuropathic pain. Angewandte Chemie International Edition 49, 6545–6548. 20533477 10.1002/anie.201000620

[CIT0009] Clemente T. 2006. Nicotiana (*Nicotiana tobaccum*, *Nicotiana benthamiana*). In: Wang K, ed. Agrobacterium protocols. Totowa, NJ: Humana Press, 143–154. 10.1385/1-59745-130-4:143 16988341

[CIT0010] Colgrave ML , Craik DJ. 2004. Thermal, chemical, and enzymatic stability of the cyclotide kalata B1: the importance of the cyclic cystine knot. Biochemistry 43, 5965–5975. 15147180 10.1021/bi049711q

[CIT0011] Conlan BF , Gillon AD, Barbeta BL, Anderson MA. 2011. Subcellular targeting and biosynthesis of cyclotides in plant cells. American Journal of Botany 98, 2018–2026. 22081413 10.3732/ajb.1100382

[CIT0012] Craik DJ , Daly NL, Bond T, Waine C. 1999. Plant cyclotides: a unique family of cyclic and knotted proteins that defines the cyclic cystine knot structural motif. Journal of Molecular Biology 294, 1327–1336. 10600388 10.1006/jmbi.1999.3383

[CIT0013] de Veer SJ , Swedberg JE, Brattsand M, Clements JA, Harris JM. 2016. Exploring the active site binding specificity of kallikrein-related peptidase 5 (KLK5) guides the design of new peptide substrates and inhibitors. Biological Chemistry 397, 1237–1249. 26894578 10.1515/hsz-2016-0112

[CIT0014] de Veer SJ , White AM, Craik DJ. 2021. Sunflower trypsin inhibitor-1 (SFTI-1): Sowing seeds in the fields of chemistry and biology. Angewandte Chemie International Edition 60, 8050–8071. 32621554 10.1002/anie.202006919

[CIT0015] Doudna JA , Charpentier E. 2014. The new frontier of genome engineering with CRISPR-Cas9. Science 346, 1258096. 25430774 10.1126/science.1258096

[CIT0016] Du JQ , Yap K, Chan LY, Rehm FBH, Looi FY, Poth AG, Gilding EK, Kaas Q, Durek T, Craik DJ. 2020. A bifunctional asparaginyl endopeptidase efficiently catalyzes both cleavage and cyclization of cyclic trypsin inhibitors. Nature Communications 11, 1575. 10.1038/s41467-020-15418-2 PMC7101308 32221295

[CIT0017] Eyraud V , Karaki L, Rahioui I, Sivignon C, Da Silva P, Rahbe Y, Royer C, Gressent F. 2013. Expression and biological activity of the cystine knot bioinsecticide PA1b (Pea Albumin 1 Subunit b). PLoS One 8, e81619. 24349099 10.1371/journal.pone.0081619 PMC3859497

[CIT0018] Fischer R , Buyel JF. 2020. Molecular farming—the slope of enlightenment. Biotechnology Advances 40, 107519. 31954848 10.1016/j.biotechadv.2020.107519

[CIT0019] Ganesan R , Dughbaj MA, Ramirez L, Beringer S, Aboye TL, Shekhtman A, Beringer PM, Camarero JA. 2021. Engineered cyclotides with potent broad in vitro and in vivo antimicrobial activity. Chemistry 27, 12702–12708. 34159664 10.1002/chem.202101438 PMC8410672

[CIT0020] Gillon AD , Saska I, Jennings CV, Guarino RF, Craik DJ, Anderson MA. 2008. Biosynthesis of circular proteins in plants. The Plant Journal 53, 505–515. 18086282 10.1111/j.1365-313X.2007.03357.x

[CIT0021] Gomez ML , Huang X, Alvarez D, et al. 2021. Contributions of the international plant science community to the fight against human infectious diseases—part 1: epidemic and pandemic diseases. Plant Biotechnology Journal 19, 1901–1920. 34182608 10.1111/pbi.13657 PMC8486245

[CIT0022] Grosse-Holz F , Madeira L, Zahid MA, Songer M, Kourelis J, Fesenko M, Ninck S, Kaschani F, Kaiser M, van der Hoorn RAL. 2018. Three unrelated protease inhibitors enhance accumulation of pharmaceutical recombinant proteins in *Nicotiana benthamiana*. Plant Biotechnology Journal 16, 1797–1810. 29509983 10.1111/pbi.12916 PMC6131417

[CIT0023] Gruis DF , Schulze J, Jung R. 2004. Storage protein accumulation in the absence of the vacuolar processing enzyme family of cysteine proteases. The Plant Cell 16, 270–290. 14688293 10.1105/tpc.016378 PMC301410

[CIT0024] Harris KS , Guarino RF, Dissanayake RS, et al. 2019. A suite of kinetically superior AEP ligases can cyclise an intrinsically disordered protein. Scientific Reports 9, 10820. 31346249 10.1038/s41598-019-47273-7 PMC6658665

[CIT0025] Higgins TJV , Chandler PM, Randall PJ, Spencer D, Beach LR, Blagrove RJ, Kortt AA, Inglis AS. 1986. Gene structure, protein-structure, and regulation of the synthesis of a sulfur-rich protein in pea seeds. Journal of Biological Chemistry 261, 1124–1130. 3755437

[CIT0026] Jackson MA , Chan LY, Harding MD, Craik DJ, Gilding EK. 2022. Data from: Rational domestication of a plant-based recombinant expression system expands its biosynthetic range. Dryad Digital Repository 10.5061/dryad.k6djh9w88 PMC9578353 35724659

[CIT0027] Jackson MA , Gilding EK, Shafee T, et al. 2018. Molecular basis for the production of cyclic peptides by plant asparaginyl endopeptidases. Nature Communications 9, 2411. 10.1038/s41467-018-04669-9 PMC6010433 29925835

[CIT0028] Jackson MA , Nguyen LTT, Gilding EK, Durek T, Craik DJ. 2020. Make it or break it: plant AEPs on stage in biotechnology. Biotechnology Advances 45, 107651. 33141031 10.1016/j.biotechadv.2020.107651

[CIT0029] Jackson MA , Yap K, Poth AG, et al. 2019. Rapid and scalable plant-based production of a potent plasmin inhibitor peptide. Frontiers in Plant Science 10, fpls.2019.00602. 10.3389/fpls.2019.00602 PMC6530601 31156672

[CIT0030] Jansing J , Sack M, Augustine SM, Fischer R, Bortesi L. 2019. CRISPR/Cas9-mediated knockout of six glycosyltransferase genes in *Nicotiana benthamiana* for the production of recombinant proteins lacking beta-1,2-xylose and core alpha-1,3-fucose. Plant Biotechnology Journal 17, 350–361. 29969180 10.1111/pbi.12981 PMC6335070

[CIT0031] Langmead B , Salzberg SL. 2012. Fast gapped-read alignment with Bowtie 2. Nature Methods 9, 357–359. 22388286 10.1038/nmeth.1923 PMC3322381

[CIT0032] Leng N , Dawson JA, Thomson JA, Ruotti V, Rissman AI, Smits BMG, Haag JD, Gould MN, Stewart RM, Kendziorski C. 2013. EBSeq: an empirical Bayes hierarchical model for inference in RNA-seq experiments. Bioinformatics 29, 1035–1043. 23428641 10.1093/bioinformatics/btt087 PMC3624807

[CIT0033] Luckett S , Garcia RS, Barker JJ, Konarev AV, Shewry PR, Clarke AR, Brady RL. 1999. High-resolution structure of a potent, cyclic proteinase inhibitor from sunflower seeds. Journal of Molecular Biology 290, 525–533. 10390350 10.1006/jmbi.1999.2891

[CIT0034] Ma JX , Ding XZ, Li ZY, Wang S. 2021. Co-expression with replicating vector overcoming competitive effects derived by a companion protease inhibitor in plants. Frontiers in Plant Science 12, fpls.2021.699442. 10.3389/fpls.2021.699442 PMC8248793 34220920

[CIT0035] Muratspahić E , Tomašević N, Koehbach J, et al. 2021. Design of a stable cyclic peptide analgesic derived from sunflower seeds that targets the κ-opioid receptor for the treatment of chronic abdominal pain. Journal of Medicinal Chemistry 64, 9042–9055. 34162205 10.1021/acs.jmedchem.1c00158 PMC8273886

[CIT0036] Nakasugi K , Crowhurst R, Bally J, Waterhouse P. 2014. Combining transcriptome assemblies from multiple de novo assemblers in the allo-tetraploid plant *Nicotiana benthamiana*. PLoS One 9, e91776. 24614631 10.1371/journal.pone.0091776 PMC3948916

[CIT0037] Nakaune S , Yamada K, Kondo M, Kato T, Tabata S, Nishimura M, Hara-Nishimura I. 2005. A vacuolar processing enzyme, delta VPE, is involved in seed coat formation at the early stage of seed development. The Plant Cell 17, 876–887. 15705955 10.1105/tpc.104.026872 PMC1069705

[CIT0038] Nandi S , Kwong AT, Holtz BR, Erwin RL, Marcel S, McDonald KA. 2016. Techno-economic analysis of a transient plant-based platform for monoclonal antibody production. MAbs 8, 1456–1466. 27559626 10.1080/19420862.2016.1227901 PMC5098453

[CIT0039] Olinger GG Jr , Pettitt J, Kim D, et al. 2012. Delayed treatment of Ebola virus infection with plant-derived monoclonal antibodies provides protection in rhesus macaques. Proceedings of the National Academy of Sciences, USA 109, 18030–18035. 10.1073/pnas.1213709109 PMC3497800 23071322

[CIT0040] Pavli OI , Kelaidi GI, Tampakaki AP, Skaracis GN. 2011. The *hrpZ* gene of *Pseudomonas syringae* pv. *phaseolicola* enhances resistance to rhizomania disease in transgenic *Nicotiana benthamiana* and sugar beet. PLoS One 6, e17306. 21394206 10.1371/journal.pone.0017306 PMC3048869

[CIT0041] Poon S , Harris KS, Jackson MA, McCorkelle OC, Gilding EK, Durek T, Van Der Weerden NL, Craik DJ, Anderson MA. 2018. Co-expression of a cyclizing asparaginyl endopeptidase enables efficient production of cyclic peptides in planta. Journal of Experimental Botany 69, 633–641. 29309615 10.1093/jxb/erx422 PMC5853369

[CIT0042] Rehm FBH , Jackson MA, De Geyter E, Yap K, Gilding EK, Durek T, Craik DJ. 2019. Papain-like cysteine proteases prepare plant cyclic peptide precursors for cyclization. Proceedings of the National Academy of Sciences, USA 116, 7831–7836. 10.1073/pnas.1901807116 PMC6475389 30944220

[CIT0043] Rehm FBH , Tyler TJ, Xie J, Yap K, Durek T, Craik DJ. 2021. Asparaginyl ligases: new enzymes for the protein engineer’s toolbox. ChemBioChem 22, 2079–2086. 33687132 10.1002/cbic.202100071

[CIT0044] Riley BT , Ilyichova O, de Veer SJ, Swedberg JE, Wilson E, Hoke DE, Harris JM, Buckle AM. 2019. KLK4 inhibition by cyclic and acyclic peptides: structural and dynamical insights into standard-mechanism protease inhibitors. Biochemistry 58, 2524–2533. 31058493 10.1021/acs.biochem.9b00191

[CIT0045] Rojo E , Martin R, Carter C, et al. 2004. VPE gamma exhibits a caspase-like activity that contributes to defense against pathogens. Current Biology 14, 1897–1906. 15530390 10.1016/j.cub.2004.09.056

[CIT0046] Sainsbury F , Thuenemann EC, Lomonossoff GP. 2009. pEAQ: versatile expression vectors for easy and quick transient expression of heterologous proteins in plants. Plant Biotechnology Journal 7, 682–693. 19627561 10.1111/j.1467-7652.2009.00434.x

[CIT0047] Saska I , Gillon AD, Hatsugai N, Dietzgen RG, Hara-Nishimura I, Anderson MA, Craik DJ. 2007. An asparaginyl endopeptidase mediates in vivo protein backbone cyclization. Journal of Biological Chemistry 282, 29721–29728. 17698845 10.1074/jbc.M705185200

[CIT0048] Serra A , Hemu XY, Nguyen GKT, Nguyen NTK, Sze SK, Tam JP. 2016. A high-throughput peptidomic strategy to decipher the molecular diversity of cyclic cysteine-rich peptides. Scientific Reports 6, srep23005. 10.1038/srep23005 PMC4786859 26965458

[CIT0049] Shimada T , Yamada K, Kataoka M, et al. 2003. Vacuolar processing enzymes are essential for proper processing of seed storage proteins in *Arabidopsis thaliana*. Journal of Biological Chemistry 278, 32292–32299. 12799370 10.1074/jbc.M305740200

[CIT0050] Slazak B , Kapusta M, Malik S, Bohdanowicz J, Kuta E, Malec P, Goransson U. 2016. Immunolocalization of cyclotides in plant cells, tissues and organ supports their role in host defense. Planta 244, 1029–1040. 27394154 10.1007/s00425-016-2562-y PMC5052299

[CIT0051] Streatfield SJ. 2007. Approaches to achieve high-level heterologous protein production in plants. Plant Biotechnology Journal 5, 2–15. 17207252 10.1111/j.1467-7652.2006.00216.x

[CIT0052] Swedberg JE , de Veer SJ, Sit KC, Reboul CF, Buckle AM, Harris JM. 2011. Mastering the canonical loop of serine protease inhibitors: enhancing potency by optimising the internal hydrogen bond network. PLoS One 6, e19302. 21556330 10.1371/journal.pone.0019302 PMC3083445

[CIT0053] Swedberg JE , Wu GJ, Mahatmanto T, Durek T, Caradoc-Davies TT, Whisstock JC, Law RHP, Craik DJ. 2019. Highly potent and selective plasmin inhibitors based on the sunflower trypsin inhibitor-1 scaffold attenuate fibrinolysis in plasma. Journal of Medicinal Chemistry 62, 552–560. 30520638 10.1021/acs.jmedchem.8b01139

[CIT0054] Tsutsui H , Higashiyama T. 2016. pKAMA-ITACHI vectors for highly efficient CRISPR/Cas9-mediated gene knockout in *Arabidopsis thaliana*. Plant & Cell Physiology 58, 46–56. 10.1093/pcp/pcw191 PMC5444565 27856772

[CIT0055] Walwyn DR , Huddy SM, Rybicki EP. 2015. Techno-economic analysis of horseradish peroxidase production using a transient expression system in *Nicotiana benthamiana*. Applied Biochemistry and Biotechnology 175, 841–854. 25344434 10.1007/s12010-014-1320-5

[CIT0056] Wang CK , Craik DJ. 2018. Designing macrocyclic disulfide-rich peptides for biotechnological applications. Nature Chemical Biology 14, 417–427. 29662187 10.1038/s41589-018-0039-y

[CIT0057] Ward BJ , Gobeil P, Séguin A, et al. 2021a. Phase 1 randomized trial of a plant-derived virus-like particle vaccine for COVID-19. Nature Medicine 27, 1071–1078. 10.1038/s41591-021-01370-1 PMC8205852 34007070

[CIT0058] Ward BJ , Séguin A, Couillard J, Trépanier S, Landry N. 2021b. Phase III: randomized observer-blind trial to evaluate lot-to-lot consistency of a new plant-derived quadrivalent virus like particle influenza vaccine in adults 18-49 years of age. Vaccine 39, 1528–1533. 33581920 10.1016/j.vaccine.2021.01.004

[CIT0059] Wong CTT , Rowlands DK, Wong CH, Lo TWC, Nguyen GKT, Li HY, Tam JP. 2012. Orally active peptidic bradykinin B-1 receptor antagonists engineered from a cyclotide scaffold for inflammatory pain treatment. Angewandte Chemie International Edition 51, 5620–5624. 22532483 10.1002/anie.201200984

[CIT0060] Wuest DM , Harcum SW, Lee KH. 2012. Genomics in mammalian cell culture bioprocessing. Biotechnology Advances 30, 629–638. 22079893 10.1016/j.biotechadv.2011.10.010 PMC3718848

[CIT0061] Yu RL , Kompella SN, Adams DJ, Craik DJ, Kaas Q. 2013. Determination of the alpha-Conotoxin Vc1.1 binding bite on the alpha 9 alpha 10 nicotinic acetylcholine receptor. Journal of Medicinal Chemistry 56, 3557–3567. 23566299 10.1021/jm400041h

