# Supplementary material for: Rational domestication of a plant-based recombinant expression system expands its biosynthetic range
Source: J Exp Bot. 2022 Jun 20;73(18):6103–14. doi: 10.1093/jxb/erac273 (PMC9578353; doi:10.1093/jxb/erac273)
Supplement: erac273_suppl_Supplementary_Material [file erac273_suppl_supplementary_material.pdf]

Supplementary data:

Rational domestication of a plant-based recombinant expression system expands its biosynthetic range.

Mark A. Jackson, Lai Yue Chan, Maxim D. Harding, David J. Craik*, Edward K. Gilding*.

Author affiliation: Institute for Molecular Bioscience, Australian Research Council Centre of Excellence for Innovations in Peptide and Protein Science, The University of Queensland, Brisbane, Queensland 4072, Australia

*Corresponding authors

e.gilding@imb.uq.edu.au or d.craik@imb.uq.edu.au

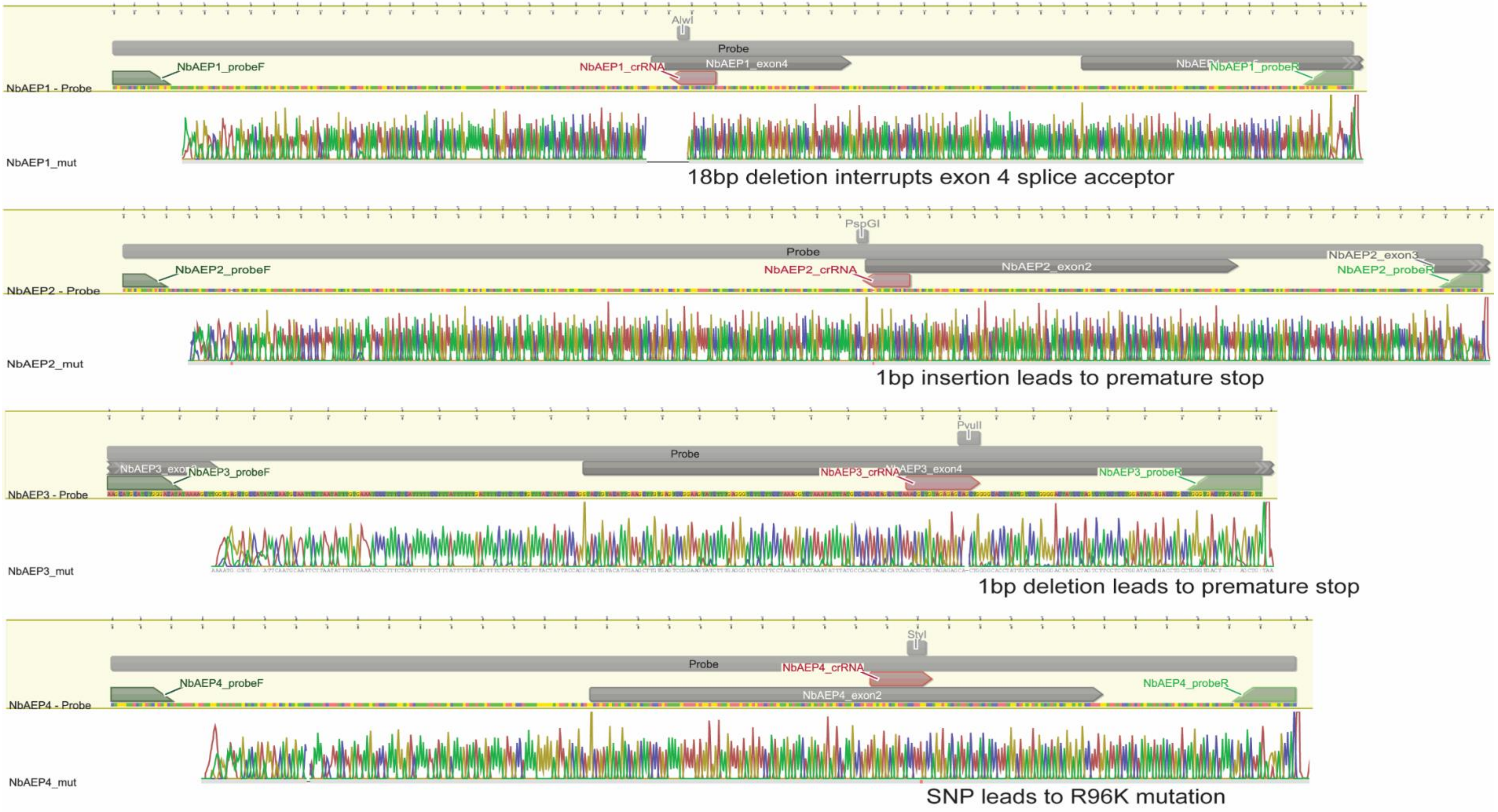

Figure S1. crRNA target sites for NbAEP1, 2, 3 and 4, CAPS marker sites, and resulting mutations for the ΔAEP genotype. NbAEP amplicons are given as grey bars labelled as probe, amplified with probeF (forward) and probeR (reverse) primers, and digested using sites annotated by grey rectangles for CAPS marker analysis. Red font and grey and red shapes denote the location of crRNA targets. Notation on the allelic change from wild-type is given under the representative Sanger sequencing trace for each locus.

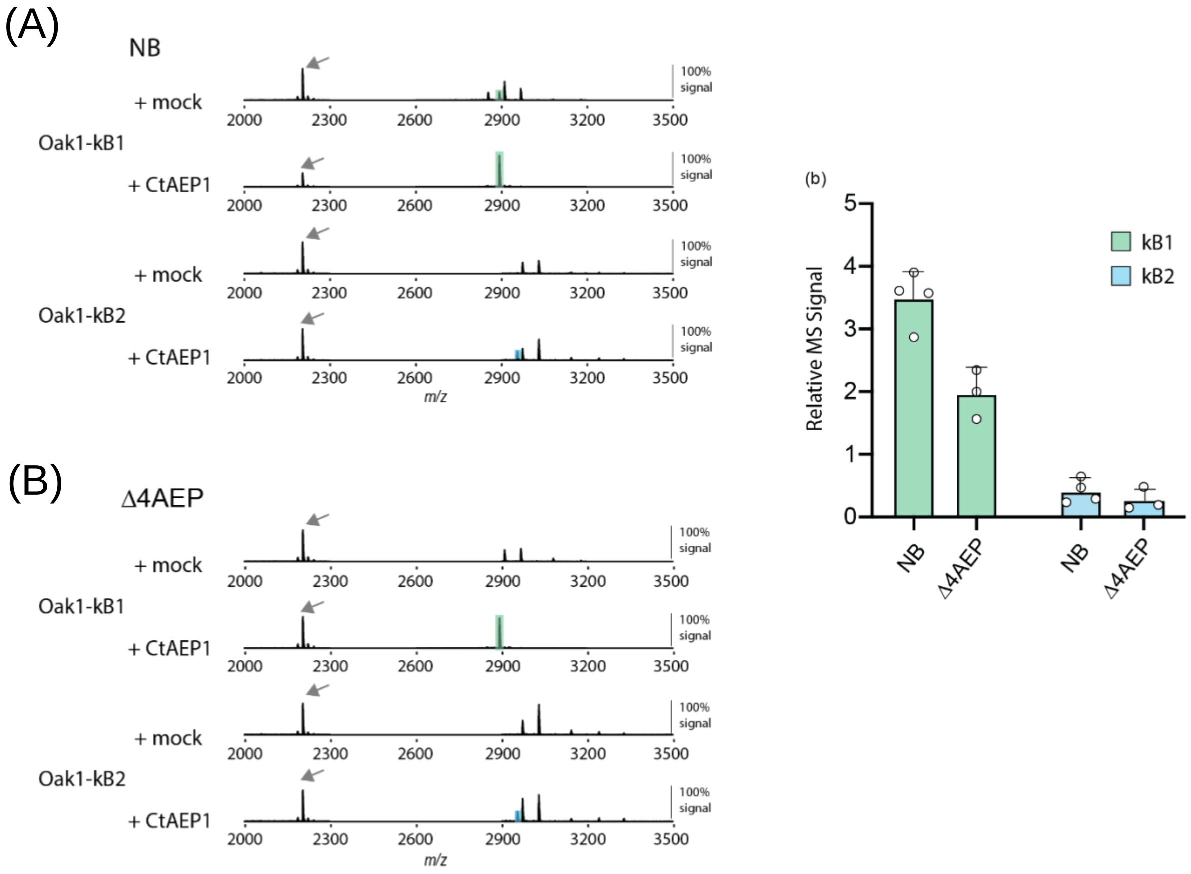

Figure S2. (A) Representative MALDI-TOF-MS of kB1 and kB2 accumulated in wild type *N. benthamiana* (NB) and the ΔAEP accession. MS signals for cyclic peptides are highlighted to match the colours in panel b. An arrow indicates the MS signal for the internally spiked peptide control that served to normalise MS signals for relative quantification. **(B)** Mean and SD (n=3 or 4) of relative kB1 and kB2 MS signals detected in crude peptide extracts of infiltrated *N. benthamiana* (NB) and the ΔAEP accession.

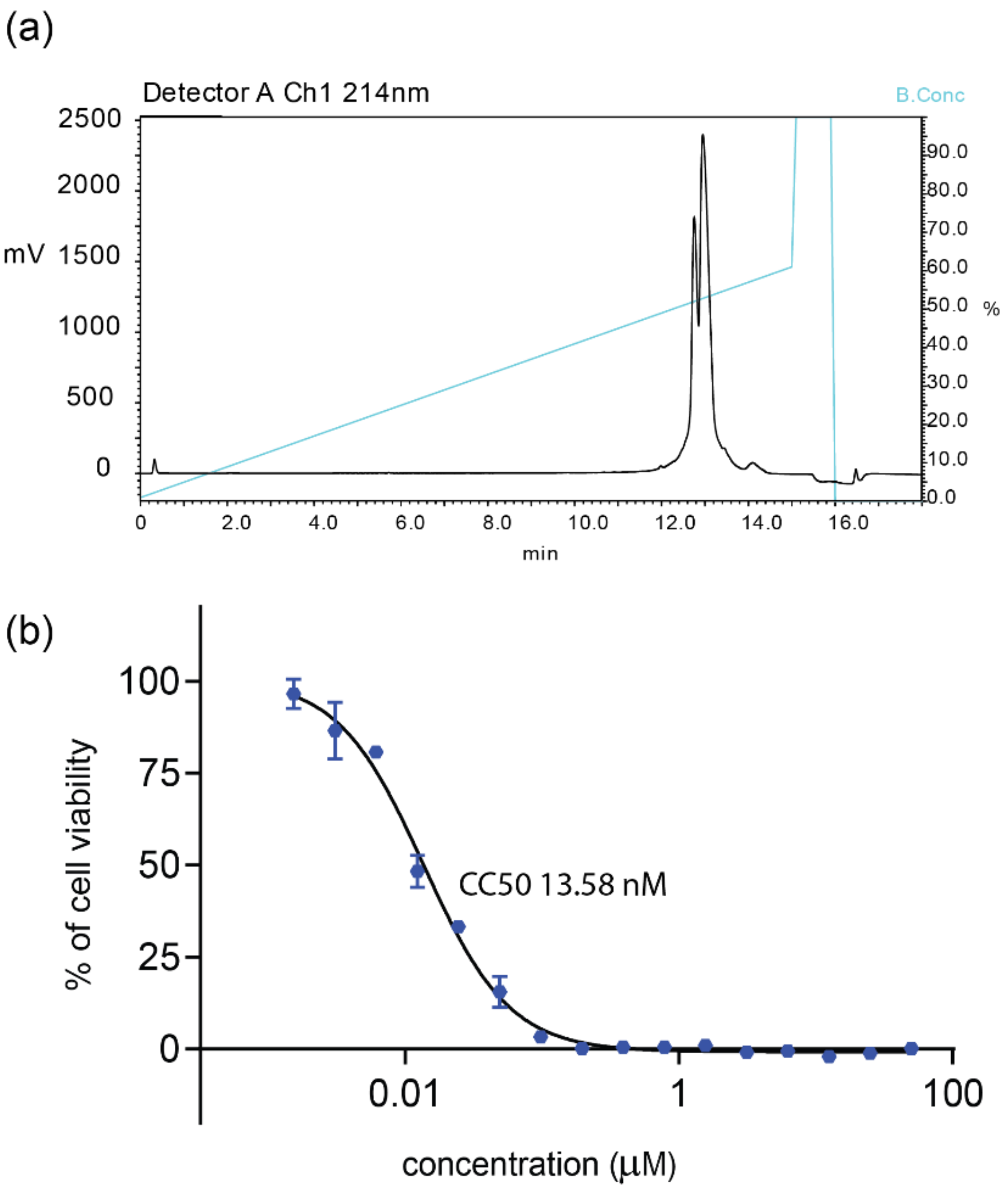

Figure S3. (a) HPLC trace of purified Pa1b. The early eluting peak was shown to be Pa1b carrying an oxidised methionine. **(b)** Cytotoxicity of Pa1b against Sf9 cells.

Oak SFTI

ATGGCTAAGTTTACCGTGTGCCTTTTGCTCTGCCTTCTCCTCGCTGCTTTTGTTGGAGCTTTCGGATCTGAGCTTTCTGATTCTCACAAGACCACCCTCGTGAACGA
GATCGCTGAGAAGATGCTCCAGAGAAAGATCCTCGATGGTGTTGAGGCTACTCTCGTGACTGATGTGGCAGAGAAGATGTTCCTCAGAAAGATGAAGGCTGAGGCTA
AGACCTCTGAGACTGCTGATCAGGTTTTCCTCAAGCAGCTTCAGCTTAAGGGAAGATGTACCAAGTCTATCCCTCCTATCTGTTTCCCTGATGGACTCCCTTCTCTT
GCTGCTTGA

MAKFTVCLLLCLLLAAFVGAFGSELSDSHKTTLVNEIAEKMLQRKILDGVEATLVTDVAEKMFLRKMKAEAKTSETADQVFLKQLQLK**GRCTKSIPPICFPD**GLPSL
AA*

Primers for SDM PCR to make Oak SFTI_N

SFTI D-N Fwd   GTTTCCCTAATGGACTCCCTTCTCTTGCTGC
SFTI D-N Rev   GTCCATTAGGGAAACAGATAGGAGGGATAGAC

Oak SFTI KLK4_D

ATGGCTAAGTTTACCGTGTGCCTTTTGCTCTGCCTTCTCCTCGCTGCTTTTGTTGGAGCTTTCGGATCTGAGCTTTCTGATTCTCACAAGACCACCCTCGTGAACGA
GATCGCTGAGAAGATGCTCCAGAGAAAGATCCTCGATGGTGTTGAGGCTACTCTCGTGACTGATGTGGCAGAGAAGATGTTCCTCAGAAAGATGAAGGCTGAGGCTA
AGACCTCTGAGACTGCTGATCAGGTTTTCCTCAAGCAGCTTCAGCTTAAGGGATTCTGTCAGAGATCTATCCCTCCTATCTGTTTCCCTGATGGACTCCCTTCTCTT
GCTGCTTGA

MAKFTVCLLLCLLLAAFVGAFGSELSDSHKTTLVNEIAEKMLQRKILDGVEATLVTDVAEKMFLRKMKAEAKTSETADQVFLKQLQLK**GFCQRSIPPICFPD**GLPSL
AA*

Primers for SDM PCR to make Oak SFTI KLK4_N

KLK4 D-N Fwd   GTTTCCCTAATGGACTCCCTTCTCTTGCTG
KLK4 D-N Rev   GTCCATTAGGGAAACAGATAGGAGGGATAG

Oak SFTI KLK5_N

ATGGCTAAGTTTACCGTGTGCCTTTTGCTCTGCCTTGTCCTCGCTGCTGTTGTTGGAGCTTTCGGATCTGAGCTTTCTGATTCTCACAAGACCACCCTCGTGAACGA
GATCGCTGAGAAGATGCTCCAGAGAAAGATCCTCGATGGTGTTGAGGCTACTCTCGTGACTGATGTGGCAGAGAAGATGTTCCTCAGAAAGATGAAGGCTGAGGCTA
AGACCTCTGAGACTGCTGATCAGGTTTTCCTCAAGCAGCTTCAGCTTAAGGGATATTGTAATAGATCTTATCCTCCTGAATGTTTCCCTAATGGACTCCCTTCTCTT
GCTGCTTGA

MAKFTVCLLLCLVLAAVVGAFGSELSDSHKTTLVNEIAEKMLQRKILDGVEATLVTDVAEKMFLRKMKAEAKTSETADQVFLKQLQLK**GYCNRSYPPECFPN**GLPSL
AA*

Oak Vc1.1

ATGGCTAAGTTTACCGTGTGCCTTTTGCTCTGCCTTCTCCTCGCTGCTTTTGTTGGAGCTTTCGGATCTGAGCTTTCTGATTCTCACAAGACCACCCTCGTGAACGA
GATCGCTGAGAAGATGCTCCAGAGAAAGATCCTCGATGGTGTTGAGGCTACTCTCGTGACTGATGTGGCAGAGAAGATGTTCCTCAGAAAGATGAAGGCTGAGGCTA
AGACCTCTGAGACTGCTGATCAGGTTTTCCTCAAGCAGCTTCAGCTTAAGGGATGTTGCTCTGATCCTCGTTGTAATTATGATCATCCTGAAATTTGCTGA

MAKFTVCLLLCLLLAAFVGAFGSELSDSHKTTLVNEIAEKMLQRKILDGVEATLVTDVAEKMFLRKMKAEAKTSETADQVFLKQLQLK**GCCSDPRCNYDHPEIC***

Oak [N9W]Vc1.1

ATGGCTAAGTTTACCGTGTGCCTTTTGCTCTGCCTTCTCCTCGCTGCTTTTGTTGGAGCTTTCGGATCTGAGCTTTCTGATTCTCACAAGACCACCCTCGTGAACGA
GATCGCTGAGAAGATGCTCCAGAGAAAGATCCTCGATGGTGTTGAGGCTACTCTCGTGACTGATGTGGCAGAGAAGATGTTCCTCAGAAAGATGAAGGCTGAGGCTA
AGACCTCTGAGACTGCTGATCAGGTTTTCCTCAAGCAGCTTCAGCTTAAGGGATGTTGCTCTGATCCTCGTTGTTGGTATGATCATCCTGAAATTTGCTGA

MAKFTVCLLLCLLLAAFVGAFGSELSDSHKTTLVNEIAEKMLQRKILDGVEATLVTDVAEKMFLRKMKAEAKTSETADQVFLKQLQLK**GCCSDPRCWYDHPEIC***

Oak_HIIAA

ATGGCTAAGTTTACCGTGTGCCTTTTGCTCTGCCTTCTCCTCGCTGCTTTTGTTGGAGCTTTCGGATCTGAGCTTTCTGATTCTCACAAGACCACCCTCGTGAACGA
GATCGCTGAGAAGATGCTCCAGAGAAAGATCCTCGATGGTGTTGAGGCTACTCTCGTGACTGATGTGGCAGAGAAGATGTTCCTCAGAAAGATGAAGGCTGAGGCTA
AGACCTCTGAGACTGCTGATCAGGTTTTCCTCAAGCAGCTTCAGCTTAAGGGACTCCCTGTTTGCGGAGAGACTTGTGTTGGAGGAACTTGCAACACTCCTGGATGC
ACTTGTTCTTGGCCTGTGTGTACTAGAAACCATATTATCGCTGCTTGA

MAKFTVCLLLCLLLAAFVGAFGSELSDSHKTTLVNEIAEKMLQRKILDGVEATLVTDVAEKMFLRKMKAEAKTSETADQVFLKQLQLK**GLPVCGETCVGGTCNTPGC
TCSWPVCTRN**HIIAA*

PA1

ATGGCTTCTGTTAAGCTTGCTTCTCTGATCGTGCTGTTCGCTACCCTTGGTATGTTCCTGACTAAGAACGTGGGTGCTGCTTCTTGCAATGGTGTGTGCTCTCCTTT
CGAAATGCCTCCTTGTGGTACTAGCGCTTGCAGATGCATTCCTGTGGGTCTTGTTGTGGGATACTGCAGAAATCCTAGCGGTGTGTTCCTGAGGACTAACGATGAGC
ATCCTAACCTGTGCGAGTCCGATGCTGATTGCAGAAAGAAGGGTTCCGGTAACTTCTGCGGTCACTACCCTAACCCTGATATCGAGTACGGTTGGTGCTTCGCTTCT
AAGTCTGAGGCTGAGGATTTTTTCAGCAAGATTACCCCTAAGGATCTGCTGAAGTCCGTGTCTACTGCTTAG

MASVKLASLIVLFATLGMFLTKNVGA**ASCNGVCSPFEMPPCGTSACRCIPVGLVVGYCRNPSG**VFLRTNDEHPNLCESDADCRKKGSGNFCGHYPNPDIEYGWCFAS
KSEAEDFFSKITPKDLLKSVSTA*

Figure S4. Gene sequences ordered as dsDNA gene blocks and primers designed for site directed mutagenesis experiments.

Table S1. pGEMT-NbAEP construction.

Product name	For-Name	Forward-Seq	Rev-Name	Rev-Seq	bp
pGEMT-MOD	pGEMT-AarI_F	TGCAGTTTTTTTGCAGGTGGGCGAATCACTAGTGCGGCCGCCTGCAGG	pGEMT-AarI_R	GTTCAATTTTTGCAGGTGGCGCTCATCCCGCGGCCATGGCGGC	3003
Nb-PROD1	pTG_5termAarI_F	CACCTGCAAAAATTGAACAAAGCACCAGTGGTCTAG	NbAEP1pTGv2_R	ACCGATCCGTACCTCTATGCTGCACCAGCCGGGAATCG	112
Nb-PROD2	NbAEP1pTGv2_F	GCATAGAGGTACGGATCGGTGTTTTAGAGCTAGAAATAGC	NbAEP2pTGv2_R	GGCAGATGTTTGTCACGCATTGCACCAGCCGGGAATCG	193
Nb-PROD3	NbAEP2pTGv2_F	ATGCGTGACAAACATCTGCCGTTTTAGAGCTAGAAATAGC	NbAEP3pTGv2_R	CAGCTGCTCTCTACAGCGTTTGCACCAGCCGGGAATCG	193
Nb-PROD4	NbAEP3pTGv2_F	AACGCTGTAGAGAGCAGCTGGTTTTAGAGCTAGAAATAGC	NbAEP4pTGv2_R	GGCCTTGGATTCAGCTCACTTGCACCAGCCGGGAATCG	193
Nb-PROD5	NbAEP4pTGv2_F	AGTGAGCTGAATCCAAGGCCGTTTTAGAGCTAGAAATAGC	pTG_3termAarI_R	CACCTGCAAAAAAACTGCACCAGCCGGGAATC	188
pTG_mid	GTTTTAGAGCTAGAAATAGCAAGTTAAAATAAGGCTAGTCCGTTATCAACTTGAAAAAGTGGCACCGAGTCGGTGCAACAAAGCACCAGTGGTCTAGTGGTAGAATAGTACCCTGCCACGGTACAGACCCGGGTTCGATTCCCGGCTGGTGCA				

Table S2. Primers for genotyping ΔAEP plants.

Product name	Expected size (bp)	Name	Sequence
NbAEP1	534	NbAEP1_probeF	TAGATATCACTGTCGTATATGGAGG
		NbAEP1_probeR	ATAGGTTCCCCAGCTACTCTC
NbAEP2	604	NbAEP2_probeF	AGTAGCGGTTTATGAGCATG
		NbAEP2_probeR	GCTGTAACATGATGCCCTG
NbAEP3	311	NbAEP3_probeF	AAGCATGCATCTGGGACATA
		NbAEP3_probeR	AACAGCATACAAGTCACCCA
NbAEP4	381	NbAEP4_probeF	CTGGGTTACTGATCGATGCT
		NbAEP4_probeR	GAGGCACCATTATTGATGCC

Table S3. Genes significantly upregulated and downregulated greater than 2-fold.

GeneID	logFC	logCPM	F	PValue	Blast-Hit-Accession	Human-Readable-Description
Niben101Scf02659g01005.1	-3.430585879	3.622925739	90.71901253	0.000195837	sp|A8H1G3|G6PI_SHEPA	Glucose-6-phosphate isomerase
Niben101Scf04539g04014.1	-3.28908874	3.947356753	121.6809836	9.55659E-05	sp|P49043|VPE_CITSI	Vacuolar-processing enzyme
Niben101Scf04675g08014.1	-2.946428049	5.849135063	64.58170801	0.000443867	sp|P49043|VPE_CITSI	Vacuolar-processing enzyme
Niben101Scf18356g00003.1	-2.913219846	4.700707579	50.47443889	0.000795079	sp|P49043|VPE_CITSI	Vacuolar-processing enzyme
Niben101Scf07493g04001.1	2.043953945	4.082535494	63.9637378	0.000454159	AT5G42830.1	HXXXD-type acyl-transferase family protein LENGTH=450
Niben101Scf04273g00001.1	2.088021671	4.87781374	7.48930441	0.040191691	sp|Q11PV4|METE_CYTH3	5-methyltetrahydropteroyltriglutamate--homocysteine methyltransferase
Niben101Scf03147g09006.1	2.288482969	4.599855983	8.415615305	0.03305199	sp|Q9SR36|GSTU8_ARATH	Glutathione S-transferase U8
Niben101Scf01789g04010.1	2.436064447	5.133006085	7.813826473	0.037463521	sp|Q15782|CH3L2_HUMAN	Chitinase-3-like protein 2
Niben101Scf01481g01006.1	2.648302619	4.772189075	28.28017701	0.002981915	sp|Q9FLS0|FB253_ARATH	F-box protein
Niben101Scf01970g01015.1	2.6541105	3.637694869	28.41805886	0.002950029	sp|Q0VBY0|CMC4_BOVIN	Cx9C motif-containing protein 4
Niben101Scf07242g01001.1	2.791907417	5.461647938	7.293642233	0.04197315	AT3G28740.1	Cytochrome P450 superfamily protein LENGTH=509
Niben101Scf01660g00007.1	2.981822846	4.909884544	7.812794329	0.037471779	AT4G14090.1	UDP-Glycosyltransferase superfamily protein LENGTH=456
Niben101Scf02217g07009.1	3.109945068	6.206317215	8.649909024	0.031530882	sp|Q4FRX3|MAO1_PSYA2	NAD-dependent malic enzyme
Niben101Scf02349g03001.1	3.394246764	7.402016387	20.95584041	0.005703932	sp|A7NY33|PER4_VITVI	Peroxidase 4
Niben101Scf01237g09002.1	3.790827628	3.3046639	51.71549607	0.000751034	sp|Q9FFB8|CHX3_ARATH	Cation/H(+) antiporter 3
Niben101Scf03886g04003.1	3.868166949	4.323765449	20.76272895	0.005816618	sp|Q9FED2|HA22E_ARATH	HVA22-like protein e
Niben101Scf14427g00009.1	5.106760145	4.85019011	19.49419786	0.00664011	0	Unknown protein
